# Comprehensive substance use services within primary care settings: The Safer Opioid Supply program at London InterCommunity Health Centre

**DOI:** 10.17269/s41997-025-01006-8

**Published:** 2025-03-12

**Authors:** Kaitlin Fajber, Andrea Sereda, Sean Warren, Cassidy Morris, Greg Nash, Bernie Pauly, Karen Urbanoski, Gillian Kolla

**Affiliations:** 1 Canadian Institute for Substance Use Research, University of Victoria, Victoria, BC Canada; 2 London InterCommunity Health Centre, London, ON Canada; 3 Memorial University of Newfoundland, St. John’s, NL Canada

**Keywords:** Overdose, Overdose crisis, Safer supply, People who use drugs, Harm reduction, Safer opioid supply, Prescribed safer supply, Primary health care, Equity-oriented care, Surdose, Crise des surdoses, Approvisionnement plus sécuritaire, Personnes faisant usage de drogue, Réduction des mesfaits, Approvisionnement plus sécuritaire en opioïdes, Approvisionnement plus sécuritaire en médicaments sur ordonnance, Soins de santé primaires, Soins promouvant l’équité

## Abstract

**Setting:**

This paper describes the Safer Opioid Supply (SOS) program, a public health intervention in London, Ontario, in response to the toxic unregulated drug supply which is driving the overdose crisis in Canada.

**Intervention:**

The London InterCommunity Health Centre (LIHC) SOS program provides comprehensive harm reduction and primary health care services to individuals at risk of overdose from the toxic drug supply. Clients are prescribed high-dose pharmaceutical opioids as replacement for unregulated toxic substances within a low-barrier primary care clinic, with wraparound interdisciplinary social services, embedded in the Ontario Community Health Centre model of care. The program serves people dependent on street-acquired fentanyl who are experiencing medical issues due to their substance use, and who are experiencing challenges accessing other forms of healthcare.

**Outcomes:**

A qualitative analysis of interviews and focus groups conducted in 2022–2023 with staff (*n*=5) and clients (*n*=20) was used to explore impacts of the SOS program. Four outcomes are discussed: safer supply as crucial to engage clients in primary care; safer supply as one component of comprehensive care; the use of a harm reduction approach; and challenges with limited medication options and program capacity.

**Implications:**

Positive health and social outcomes demonstrate the utility of embedding comprehensive substance use services within a primary health care model to address health and social complexity among people who use drugs amid the continuing toxic drug crisis. Responding to an increasingly volatile unregulated supply of drugs, having limited medication options, and providing comprehensive care without long-term funding remain ongoing challenges.

## Résumé

**Lieu:**

Notre article porte sur le Programme d’approvisionnement plus sécuritaire en opioïdes (APSO), une intervention de santé publique menée à London (Ontario) en riposte à l’approvisionnement non réglementé en drogues toxiques qui alimente la crise des surdoses au Canada.

**Intervention:**

Le programme APSO du London InterCommunity Health Centre (LIHC) fournit des services intégrés de réduction des méfaits et de soins de santé primaires aux personnes à risque de faire des surdoses, car elles ont recours à l’approvisionnement en drogues toxiques. Dans une clinique de soins primaires à bas seuil offrant des services sociaux interdisciplinaires complets, selon le modèle ontarien des centres de santé communautaire, ces personnes se font prescrire de fortes doses d’opioïdes pharmaceutiques pour remplacer les substances toxiques non réglementées. Le programme sert les personnes dépendantes du fentanyl acheté dans la rue qui éprouvent des problèmes de santé causés par leur consommation et qui ont du mal à obtenir d’autres formes de soins de santé.

**Résultats:**

Pour explorer les effets du programme APSO, nous avons procédé à l’analyse qualitative des données d’entretiens et de groupes de discussion menés en 2022-2023 avec le personnel (n=5) et les usagères et usagers (n=20) de la clinique. Nous présentons quatre résultats: l’importance cruciale de l’approvisionnement plus sécuritaire pour fournir des soins primaires aux usagères et aux usagers; l’approvisionnement plus sécuritaire comme élément de soins intégrés; l’emploi d’une démarche de réduction des méfaits; et les difficultés posées par le peu d’options de médicaments et la capacité limitée du programme.

**Conséquences:**

Les effets sanitaires et sociaux positifs montrent qu’il est utile, compte tenu de la complexité des besoins sociaux et de santé des personnes faisant usage de drogue dans le cadre de la crise des drogues toxiques actuelle, d’intégrer des services d’usage de substances complets dans un modèle de soins de santé primaires. Il demeure toutefois difficile de riposter à l’approvisionnement non réglementé de plus en plus instable avec des options de médicaments limitées et de fournir des soins complets en l’absence de financement à long terme.

## Introduction

The high rates of deaths due to drug toxicity overdoses in Canada are a result of an increasingly volatile and toxic drug supply consisting primarily of unregulated fentanyl, fentanyl analogues, and increasingly, benzodiazepines (Government of Canada, 2022a, b; Russell et al., 2023). Specifically in London, Ontario, deaths due to opioid toxicity have been rising since fentanyl entered the unregulated drug market in 2016 (Public Health Ontario, 2022). In 2021, there was a fourfold increase in the number of deaths from opioid toxicity compared to 2016; across the province of Ontario, 83.5% of opioid-related deaths had fentanyl present in 2022 (Public Health Ontario, 2022). Simultaneously, the housing crisis in London persists. In early 2024, there were an estimated 1700–2100 people experiencing homelessness, with local government strategizing to accelerate building affordable and supportive housing units (Lacasse, 2023; LeBel, 2024). People who use drugs (PWUD) navigate risks associated with using unregulated substances while also frequently living at the nexus of compounding public health crises: escalating drug toxicity, the lack of affordable and accessible housing, and inequitable access to health care. In response, safe supply—access to a regulated and legal supply of substances—has been proposed as a critical intervention to uphold human rights and save lives of PWUD (Bonn et al., 2020; Canadian Association of People who Use Drugs, 2019; Tyndall, 2020).

The London InterCommunity Health Centre (LIHC) Safer Opioid Supply (SOS) program is a primary care-based model for providing prescribed safer supply to individuals at risk of overdose from the toxic drug supply. SOS program clients receive individualized prescriptions for SOS medications as one element of the harm reduction and primary health care services available to them in a low-barrier, community health centre (CHC) setting. Staff provide SOS prescriptions alongside primary health care while also advocating to improve equity-oriented care for PWUD as part of a community response to intersecting health and social crises.

In this paper, we explore the utility of embedding comprehensive substance use services within a primary health care model to address both the health of PWUD and the continuing toxic drug supply crisis. First, we describe the rationale and genesis of the SOS program, which prescribes high-dose pharmaceutical opioids to people dependent on the toxic unregulated drug supply within a primary care clinic and alongside wraparound interdisciplinary supports, using a trauma-informed care lens. Second, we describe program implementation and operation following receipt of Health Canada funding in 2020. Third, we draw on interviews with clients and staff to discuss the outcomes of providing a prescribed safer supply within a primary care setting. Finally, we discuss how the SOS program can serve as a model for the provision of comprehensive substance use services within primary care, filling a gap in the spectrum of substance use supports and enhancing equitable access to care.

## Methods

We used data collected as part of ongoing research with LIHC’s SOS program to explore the outcomes of embedding a SOS program within a primary care model within a CHC. Separate interview guides for staff and clients were developed in collaboration with an advisory group of SOS clients to ask questions about the local drug supply, a variety of program impacts on health and social outcomes, staff roles (staff only), reasons for involvement, and experiences in the program (clients only). Semi-structured interviews and focus groups were conducted in-person by GK with clients (*n*=20; 9 clients participated in individual interviews and 11 clients participated in three focus groups) in June 2022. SOS clients were invited to participate while onsite at LIHC for clinic visits at the end of their appointment and received an honorarium ($25) for participating; interviews and focus groups lasted from 21 to 64 min. Semi-structured interviews with SOS program staff (*n*=5) were conducted over video conference (Zoom) by GK and BP between November 2022 and February 2023 and lasted 46 to 68 min. Staff were recruited through an email invitation sent by research staff with an invitation to contact researchers directly if they wished to participate. All interviews were audio-recorded, transcribed, reviewed to remove potentially identifying information, and uploaded to NVivo for coding. Thematic analysis was used to identify core themes and ideas.

## Intervention

### Rationale for embedding comprehensive substance use services in primary care and program implementation

LIHC is a longstanding CHC (est. 1989) offering various health services and targeted programs to meet the needs of marginalized and vulnerable community members. The SOS program was initiated in 2016 by a primary care physician (AS) at LIHC who worked primarily with clients engaged in survival sex work and/or experiencing homelessness and precarious housing. She observed how some clients would cycle through using the unregulated supply, being hospitalized due to related harms like overdose and infectious complications, stabilizing on prescribed opioids while in hospital, and then being discharged—only to repeat this cycle again. To interrupt this cycle and reduce harms, she extended the opioid prescriptions for three clients following discharge from the hospital; stabilizing them on a prescription of take-home hydromorphone tablets to replace their use of opioids from the unregulated markets allowed for treatment of other unaddressed health needs. This included provision of harm reduction services (e.g., sterile drug use equipment and naloxone distribution) alongside primary care.

The program slowly expanded from 2016 to 2020, when LIHC received funding from Health Canada’s Substance Use and Addiction Program (SUAP) to expand their SOS program. This funding allowed LIHC to increase staff (particularly nursing and social support staff) and expand enrollment of eligible clients; this doubled their client base from 112 clients as of April 1, 2020, to 248 clients as of September 2021 (Kolla et al., 2021). Following receipt of SUAP funding, the SOS program became a specialized program at LIHC with formal program evaluation. As of March 31, 2024, the program was providing care to almost 300 clients, who all receive primary care, as well as comprehensive substance use services including individualized prescriptions of SOS and/or opioid agonist therapy (OAT) medications, access to social supports (e.g., community workers, care facilitators) and systems navigators, access to harm reduction education and equipment, and referrals to specialist medical care and treatment supports where necessary. Research and evaluation has documented outcomes specifically from the LIHC SOS program, including reductions in overdose, reduction in use of drugs from the unregulated market and use of drugs by injection, and significant decreases in emergency department use and hospitalizations among safer supply participants (Gomes et al., 2022; Kolla et al., 2021; Kolla & Fajber, 2023). Research and evaluations of prescribed safer supply programs across Canada have also described improvements in health outcomes and social stability among clients of safer supply programs (Atkinson, 2023; Gagnon et al., 2023; Haines & O’Byrne, 2023; Henderson et al., 2024; Kalicum et al., 2024; Kolla et al., 2024; Ledlie et al., 2024; McCall et al., 2024; McMurchy & Palmer, 2022; Nafeh et al., 2023; Perri et al., 2023; Schmidt et al., 2023a, b, 2024), as well as significant reductions in both overdose and all-cause mortality among people prescribed safer supply (Slaunwhite et al., 2024).

### Program operations

The SOS program prescribes safer supply medications (primarily immediate-release hydromorphone tablets) as pharmaceutical alternatives to a toxic unregulated drug supply, alongside traditional OAT medications like methadone, buprenorphine and slow-release oral morphine (Gomes et al., 2022; Ledlie et al., 2024). The target population are individuals at high risk of overdose and other health harms associated with drug use. Many are unhoused or unstably housed with limited access to traditional health care. Operating out of the CHC, all SOS clients are rostered as primary care patients, providing access to primary care from physicians and nurse practitioners, as well as social services from an interdisciplinary care team of social workers, care facilitators, and community workers (see Table 1).

**Table 1:** Health and social services provided within the primary care embedded SOS program

Health services	• Comprehensive primary care • Testing and treatment of blood borne infections (i.e., HIV, hepatitis C) • Sexual health care and screenings • Obstetrical and gynecologic care • Preventive care (i.e., cancer screenings and vaccinations) • Acute care • Wound care • Referrals to specialists
Social services	• Harm reduction education and access to equipment and supplies • Assistance accessing food programs and other basic needs (e.g., hygiene supplies, clothes) • Identification (ID) clinics to support access to other services • Housing support (i.e., help finding housing or to prevent housing loss) • Assistance with transportation to and accompaniment for appointments • System navigation and advocacy for clients (i.e., accessing income supports, applying for disability supports, or in the justice system) • Community outreach • Counselling • Referrals to withdrawal management and residential treatment

Findings from research on the LIHC SOS program have found that it successfully engages individuals experiencing high levels of health and social complexity, including high rates of prior OAT prescriptions, HIV and hepatitis C infection, and previous hospitalizations for infectious complications (Gomes et al., 2022). As demand on the program is large (Kolla et al., 2021), there is an equity-based approach for admitting new clients. Prospective program clients are triaged according to health and social acuity, with a focus on ensuring that both health (i.e., frequent overdose, recent hospitalization for infectious complications) and social determinants of health are taken into account, and that women, individuals involved in street-based sex work, individuals with untreated HIV, and individuals experiencing homelessness are prioritized for program access.

Once admitted to the SOS program, clients are seen two to three times per week until their medication doses are stabilized. Following stabilization, clients are typically assessed by an SOS nurse and seen by their primary care provider at LIHC on a weekly or biweekly basis, where they receive comprehensive primary care and have their medication doses adjusted if necessary. Most clients are prescribed immediate-release hydromorphone tablets (i.e., Dilaudid) in combination with long-acting opioids (most commonly slow-release oral morphine or methadone). Most clients receive daily-dispensed medications, with long-acting opioids provided as witnessed doses at pharmacies, and hydromorphone provided as take-home doses.

Clients are also assigned a care facilitator who can support them to address key social determinants of health like obtaining identification, and accessing housing, income supports (e.g., Ontario Works, Ontario Disability Support Program), employment, and educational programs. To reduce barriers to access, the LIHC also has a mobile program—Health Outreach Mobile Engagement (HOME)—which provides health care services, including SOS, on an outreach bus that visits various community locations and encampments.

## Outcomes

Our thematic analysis identified four themes which explored the utility of SOS offered as part of comprehensive primary health care. Below, the following four themes are explored: (1) safer supply as crucial to engage clients in primary care; (2) safer supply as one component of comprehensive care; (3) safer supply delivered through harm reduction approach; and (4) challenges with limited medication options and program capacity.

### Safer supply as crucial to engage clients in primary care

At the time of program entry, clients were reliant on unregulated fentanyl, and many were engaged in survival sex work, street hustles, or small-scale criminal activity to obtain drugs and/or money to obtain drugs to address withdrawal or treat pain (please see Table 2 for all quotations for the Outcomes section). Clients described how receiving a regular prescription of pharmaceutical opioids of known dose allowed them to cease these activities. Instead, clients were able to focus on activities and relationships that were important to them. As clients shifted away from the unregulated drug supply and onto SOS medications, they experienced few to no overdoses—meeting the primary aim of the program to prevent overdose deaths. Staff identified that SOS medications enabled clients to access and stay engaged in their health care. Additionally, regular primary care visits due to SOS program participation allowed clients to address both physical and mental health care needs, and on-site social services led to improvements in financial, food, and housing security.

**Table 2:** Select quotations from LIHC clients and staff

Safer supply as crucial to engage clients in primary care	Clients	“It’s [safer supply] honestly working a lot better than how I was before. You know, having to do things that I didn’t want to do or, just the stuff I had to resort to in order just to get unsick. And now, I have no reason to do anything illicit or illegal or anything.” (LIHC client, interview) “I would never go to the doc, like I probably wouldn’t go to the doctor. Like, I don’t go to, haven’t been to the doctor in years. You know what I mean? So, yeah. Probably seeing the doctor regularly helps.” (LIHC client, focus group) “It gives me back my life. I can go do things.” (LIHC client, interview)
	Staff	“We have some folks who have never engaged with a medical system before, I would say sometimes in their whole life. And having a person, you know, engaged in a medicalized model of safe supply where they come into a medical clinic, they meet with a nurse, they talk about their substance use as well as their physical primary care, chronic or acute medical concerns, and then see a doctor regularly, I think that engagement with the medical system is such a great way for us to help our folks treat some of those really important and really life threatening ailments like sepsis. Like abscess. Like endocarditis.” (LIHC Staff member) “The overall, overarching feedback we get from people is, ‘You’re the only ones who give a shit about us’. ‘We know we can come here and we know that we can tell you whatever we need to tell you and we’re not going to be judged’. [...] And certainly safe supply gave us a foot in the door that I think people feel like we’re actually addressing their needs as they state what their needs are. And that allowed us to build the trust.” (LIHC Staff member) “The fact that we’re saying street supply is not ideal but the only option isn’t just stop using it. Like that, that sets us apart. [...] But at the end of the day it’s the clients coming here. The clients choosing to participate. And the clients choosing to participate with us. You could have people come in saying: I don’t want to talk to you. I don’t want anything else. Give me my D’s [Dilaudids]. I’m gonna go. And people don’t do that. Like, people come back. People could very easily choose to not engage.” (LIHC Staff member)
Safer supply as one component of comprehensive care	Clients	“I never had a doctor period. I had so much health issues it was crazy. It just, it saved my life in EVERY aspect. From showering to eating.” (LIHC client, interview) “It was life changing. From being on the streets hooking, doing all that stuff, to going like, I started living in a place.” (LIHC client, focus group) “Just like structure and belonging, I think. And, dealing with my pain is a big one. Because when you have to deal with physical and emotional pain and psychological pain, like everything, it’s all way too much. Especially being homeless, also, at the time. Right? Just it becomes too much. Right? […] So it [the SOS program] literally saved my life.” (LIHC client, interview)
	Staff	“We had somebody yesterday, they’re housed and they’re a completely different person. Like: ‘I had to be this, this specific person when I was living on the streets and now I can be myself. Like, when I have a house and I no longer have to be like, fight or flight all the time’. I’m seeing the stark personality changes of people actually being able to just be their authentic selves without having to figure out: ‘How do I just get this? How do I meet my very, very basic needs’?” (LIHC Staff member) “We kind of work both in the medical lens as well as the social lens. In really trying to support one of our safe supply participants to tackle things like transportation, income support, housing. We work with folks who are criminalized, who may work in either survival or different types of sex work. And so our role is really to craft out goals and care plans and match a person’s care to their goals and what they’re looking for.” (LIHC Staff member) “So we are a [community health centre]. Within that we have, like we’re a full multidisciplinary clinic. So on top of primary care, we do have case facilitation dedicated to our [safer supply] program which is a case manager. We have systems navigation and within our [CHC] we have access to social work, psychiatry, physiotherapy, dietician, foot care. We have infectious disease teams on staff for Hep C and HIV-AIDS. So they have access to absolutely all of that within the centre. Our [safer supply] program is just one, one tool that we use within our primary care lens.” (LIHC Staff member)
Safer supply delivered through harm reduction approach	Clients	“It’s the best place I’ve ever been. I feel like this is my home. Like, I come in here, they know me. They know my name. You know? [...] It just makes me feel like they give a shit. They’re not just pumping me full of drugs and getting me the fuck outta here. It makes me want to be a better person, you know?” (LIHC client, interview) “I think [my SOS prescriber is] definitely a lot more willing to work with you. Like, with the methadone recently you go in, they’ll be like: “Hey, you wanna go up? Or, you wanna…. What do you wanna do?” And then they just, you just tell them what and they just prescribe you when you go. [My SOS prescriber] actually asks other things like: “How’s your living situation?” And actually care more it seems like.” (LIHC client, interview) P: I like the nurses here and everything[…]And they do help, they’re helpful. Like, those girls are just as good as the doctor, I think [...] They’ve all been good to me, yeah. (LIHC client, interview)
	Staff	“And that’s what I see. It goes far beyond just the substance replacement. It’s a different way of engaging with other human beings. [...] I think that safer supply offers, yes, a substance replacement, but if done right, it offers that in a loving and supportive way. So it’s not just replacing the opioids it’s replacing that this is lacking.” (LIHC Staff member) “I think our approach to client care works really well. I think that our model of care works really well. That harm reduction client focused care. That they lead their own health care. Their own social care. They identify what they feel their needs and wants are. And we help them with that, as they’re ready to approach each task, or step in their life. I think that works really well.” (LIHC Staff member) “Whereas here, people trust me enough to say: ‘I don’t like that you did this’. Or, I would like this. Or, people end up disclosing things. And I can help people because they say: I trust you. Let me tell you about this problem I’ve been having for a while and haven’t been able, haven’t, I’ve just been too anxious or afraid to say. So we’re actually able to help because our clients let us help them.” (LIHC Staff member)
Challenges with limited medication options and program capacity	Clients	“Cause once you get to fentanyl it’s almost like nothing else works for them. Like, seems like that. You know? Seems like the end of the rope, you know? Like the road there. It’s like you can’t get anything that’s gonna make you feel better. So, what do you do, right? So I think a safe supply of it [fentanyl], yeah. It would help them.” (LIHC client, interview) “I find a lot of people that don’t poke as much. Like, I used to know a lot of people that would smash [inject], you know what I mean? Like I, myself do. And now, a lot of the people that I used to use with are now just smoking it.” (LIHC client, focus group)
	Staff	“I think medication legislation is a barrier to us. That’s what is no longer working well where it once did. Having access to other medications and alternative medications would help us scale up in a really large way.” (LIHC Staff member) “Yeah, I describe it to my patients that they’re drinking their eighty proof whiskey and all I have is Bud Light for them, right?” (LIHC Staff member) “There’s been situations where people have had very valid medical concerns and then they’re also asking about the program. And when you say no to the program, they’re like: ‘Well, now I’m just gonna walk out with my chest pain and not get it seen’. And it’s just, it’s so unfair to people. But also it would be unfair if we took people on and then other people would suffer because we don’t have capacity. It’s a never ending cycle, but I think that’s the main thing of how we cannot, there’s still a big population that we’re missing and we can’t address.” (LIHC Staff member)

### Safer supply as one component of comprehensive care

Importantly, SOS medications were only one component of a comprehensive suite of services and supports available at LIHC to care for individuals with complex health needs. Clients discussed their complex living circumstances and how the program impacted them positively in a multitude of ways: from helping with pain management to housing stability to addressing long-standing health issues. By providing SOS prescriptions alongside comprehensive primary care, health and social care providers built relationships with SOS clients that allowed them to address multiple health and substance use-related needs. They also established individualized substance use care goals; while some clients sought abstinence, others focused on making other positive changes to their substance use, including reductions in use of street-acquired substances, stabilization on prescribed medications, engaging in medical treatment (e.g., wound care or for chronic health conditions), reconnecting with family, returning to school, or maintaining employment. Staff highlighted the varied medical and social needs of clients, with substance use being just one aspect of clients’ lives and SOS medications as one of many supports available to each client, depending on individual need.

### Safer supply delivered through harm reduction approach

Beyond the pharmaceutical effects of the medications themselves, the harm reduction approach was emphasized as critical to client success. Clients stated that LIHC staff cared about them and showed understanding about their lives beyond their use of substances; this was contrasted with frequent previous experiences of stigma and discrimination within the health care system. Clients described the importance of comprehensive care: from the moment they enter the door, care was facilitated by a team of front desk staff, nurses, and outreach and care facilitators, in addition to prescribers. Staff recognized that the program was a source of consistency for clients who were unhoused or precariously housed while also navigating a constantly changing and volatile drug supply. Staff attempted to offer client-led harm reduction by asking clients about their goals for participating in the program, and acting on client feedback to improve their care. This built trusting relationships between staff and clients over time. High retention and continued client engagement were seen as indications of the safety and trust fostered within the program.

### Challenges with limited medication options and program capacity

Clients and staff highlighted two structural factors limiting SOS program impact: limited SOS medication options and the lack of long-term program funding. Some clients found that hydromorphone was not strong enough to replace unregulated fentanyl. Additionally, many clients smoke rather than inject opioids, and the lack of smokeable options was frequently cited as a limitation. Staff highlighted that prescribing options were limited by the provincial drug formulary, which lacked coverage for other high-dose opioids and smokeable options. Use of unregulated fentanyl from the street supply has led to high opioid tolerance, and there remains a need for improved pharmaceutical-grade alternatives to the street supply of fentanyl. Another major challenge was the lack of program capacity, which limited enrollment and meant that many eligible individuals in the community were unable to enroll in the SOS program. Instead, program staff noted having to turn away eligible candidates for the SOS program, and who may have been treated for various other health conditions simultaneously, because program resources were already stretched to the maximum. This was exacerbated by restricted program funding as short-term pilot funding from the federal government was due to expire in 2025, and a lack of funding commitments from the provincial government made long-term program planning challenging.

## Discussion

The LIHC SOS program embeds prescribed safer supply as a harm reduction intervention to facilitate provision of comprehensive substance use services within a primary care setting. Access to interdisciplinary supports such as system navigators and care facilitators provides wrap-around care to a population who were previously disconnected from the health care system, and who were experiencing significant health and social complexity due to their substance use. Analysis suggests that the positive outcomes observed among SOS clients were due to a combination of being prescribed SOS medications, which enables initial and ongoing engagement in care, and interdisciplinary care delivery, which addresses social determinants of health and centers harm reduction principles of client dignity and autonomy alongside the provision of primary care.

Our findings align with other research on prescribed safer supply that demonstrated reduced overdose frequency and associated harms, improved health outcomes, and improved stability among safer supply clients (Gagnon et al., 2023; Gomes et al., 2022; Ledlie et al., 2024; Schmidt et al., 2023a; Slaunwhite et al., 2024). However, while prescribed safer supply programs across Canada share key characteristics such as the prescribing of regulated pharmaceutical alternatives to the unregulated drug supply, the program structure and services offered differ significantly across communities. Our analysis suggests that embedding prescribed safer supply within settings that provide full-scope primary care and comprehensive social supports is an effective program model for engaging clients experiencing significant health and social challenges due to their substance use, and may be adapted to other settings and local contexts.

The SOS program has continually adapted in response to community needs and available resources. Yet several challenges persist; staff continue to advocate for expanded medication options on the provincial formulary—in particular, smokeable medication options which would better reflect the shift to smoking fentanyl seen among LIHC clients and their preferences for smokeable options (Kolla et al., 2021; Kolla & Fajber, 2023; McCall et al., 2024; Pauly et al., 2024; Xavier et al., 2023). While federal SUAP funding facilitated rapid program expansion, this pilot funding was short-term (4 years of funding, later extended to include a fifth year). The reticence of provincial health systems to fund prescribed safer supply programs within primary care settings in Ontario has led to challenges in long-term program stability despite research demonstrating positive outcomes along all dimensions of the quadruple aim to improve health care for clients, staff, and the greater health system (Government of Canada, 2022b). Long-term funding uncertainty has also been stressful and challenging for staff and partner agencies who are attempting to meet client needs with limited resources, leading to staffing challenges which in turn negatively impacted client care. Safer supply programs across Canada have experienced similar challenges with retaining staff, supporting clients, and expanding program capacity without reliable long-term funding (Karamouzian et al., 2022; McCall et al., 2024).

Embedding prescribed safer supply within primary care settings allows for provision of comprehensive substance use care that centers individualized treatment of clients’ health and social needs, while also contributing to a larger public health response to the toxic drug crisis. The SOS program offers harm reduction and trauma-informed care which are foundational to equity-oriented primary care (Browne et al., 2015). Recognition of the need for broader system-level change has led LIHC to collaborate with local agencies and hospitals in the development of an ecosystem approach to address the multiple public health crises of homelessness, drug toxicity overdose deaths, and inequitable access to health care in London (Lokko & Avor, 2024). This includes the “Whole of System Community Response” plan among municipal, hospital, and community partners in London to build capacity to support people experiencing homelessness and who use drugs, including the scale-up of “Hubs” to offer comprehensive services to help people experiencing entrenched homelessness access supports and become sustainably housed (Health and Homelessness in London - Whole of Community System Response, 2024). Key to the London Hub model is the integration of comprehensive health and social care, harm reduction interventions, and substance use services across the sector, while strengthening connections between local service providers and targeting structural barriers to service access among PWUD.

## Conclusion

The SOS program at LIHC provides a model for embedding prescribed safer supply within primary care settings to provide access to comprehensive care amid the continuing drug toxicity crisis. While ongoing public health research examining the outcomes and unintentional impacts of prescribed safer supply is necessary (Nielsen et al., 2024), our analysis suggests that embedding prescribed safer supply into primary care settings is a harm reduction approach that engages individuals experiencing medical and social complexity in care that meets their needs. Prescribed safer supply models can be implemented rapidly, while also responding to local community context (Macevicius et al., 2023; Pauly et al., 2024). The SOS program provides a model for comprehensive substance use care focused on enhancing equity-oriented primary care and strengthening the continuum of services available to people who use drugs.

## Implications for policy and practice

What are the innovations in this program?

- Embedding SOS prescribing within primary care at a community health centre allowed for provision of comprehensive health and social care to a population experiencing entrenched homelessness and medical and social complexity, and who had difficulty accessing other health care and substance use supports.
- Providing SOS stabilized clients’ substance use and increased their ability to work towards their individually defined goals.
- LIHC is part of a collaborative effort among community partners to provide comprehensive care through the London Hub model, as well as integrated primary health care teams in other settings.

What are the burning research questions for innovation?

- How can expanded prescription medication options (including smokeable and injectable options) improve the care provided to people who use drugs?
- How can a wide range of options, including comprehensive substance use care that includes prescribed safer supply, be integrated with non-medicalized and community-led SOS models to improve accessibility of harm reduction services?
- How can comprehensive substance use care be integrated within other health and social care settings (e.g., hospital, emergency department, supportive housing units) to enhance the health supports available to people and ensure continuity of care?

## Data Availability

The datasets generated and analyzed during the current study are not publicly available given the sensitive nature of the research topic, as they contain confidential information that could compromise participant confidentiality and consent.
